# Structure of the human MLH1 N-terminus: implications for predisposition to Lynch syndrome

**DOI:** 10.1107/S2053230X15010183

**Published:** 2015-07-28

**Authors:** Hong Wu, Hong Zeng, Robert Lam, Wolfram Tempel, Iain D. Kerr, Jinrong Min

**Affiliations:** a Structural Genomics Consortium, University of Toronto, 101 College Street, Toronto, ON M5G 1L7, Canada; b Myriad Genetic Laboratories Inc., 320 Wakara Way, Salt Lake City, UT 84108, USA; c Department of Physiology, University of Toronto, Toronto, ON M5G 1L7, Canada

**Keywords:** ATPase, GHKL, Lynch syndrome, mismatch repair

## Abstract

Mismatch repair prevents the accumulation of erroneous insertions/deletions and non-Watson–Crick base pairs in the genome. Pathogenic mutations in the *MLH1* gene are associated with a predisposition to Lynch and Turcot’s syndromes. Although genetic testing for these mutations is available, robust classification of variants requires strong clinical and functional support. Here, the first structure of the N-terminus of human MLH1, determined by X-ray crystallography, is described. The structure shares a high degree of similarity with previously determined prokaryotic *MLH1* homologs; however, this structure affords a more accurate platform for the classification of *MLH1* variants.

## Abstract 2

The crystal structure of the human MLH1 N-terminus is reported at 2.30 Å resolution. The overall structure is described along with an analysis of two clinically important mutations.

## 1. Introduction   

Pathogenic mutations in the DNA mismatch-repair gene *MLH1* (MutL homolog 1) are associated with a predisposition to Lynch syndrome (Bronner *et al.*, 1994 ▸; Papadopoulos *et al.*, 1994 ▸), a hereditary cancer syndrome that accounts for 2–4% of all colorectal cancer cases in the US (Aaltonen *et al.*, 1998 ▸; Hampel *et al.*, 2005 ▸, 2008 ▸; Lynch & de la Chapelle, 2003 ▸). Mismatch repair (MMR) is a complex, multicomponent process that is coordinated by a number of distinct DNA-repair factors. *MLH1* homologs are conserved across all domains of life and are essential components of MMR (Lin *et al.*, 2007 ▸). Human MLH1 (hMLH1) is a 756-amino-acid, 84 kDa protein that can be roughly divided into two halves: an N-terminal domain (NTD), where the ATPase activity resides, and a C-terminal domain (CTD), which is the site of dimerization with MLH1 paralogs (Guerrette *et al.*, 1999 ▸). In higher eukaryotes, the MLH1 and PMS2 (postmeotic segregation increased 2) paralogs form a heterodimeric complex, MutLα. Once a lesion has been identified and isolated by the MutS mismatch-recognition complex, MutLα is recruited (Fukui, 2010 ▸; Martín-López & Fishel, 2013 ▸) and, *via* its C-terminal endonuclease activity (Kadyrov *et al.*, 2006 ▸), generates nicks in the heteroduplex 3′ and 5′ to the mismatch that facilitate excision and replicative repair (Kadyrov *et al.*, 2006 ▸, 2007 ▸; Modrich, 2006 ▸). While other roles for MutLα have been proposed, these are less well understood (Her *et al.*, 2002 ▸; Liu *et al.*, 2010 ▸; McVety *et al.*, 2005 ▸; Pedrazzi *et al.*, 2001 ▸; Yanamadala & Ljungman, 2003 ▸). Whilst the exact details remain unclear, the ability of MLH1 to interact with adenine nucleotides is an important factor in MMR, inducing large conformational changes in the protein (Sacho *et al.*, 2008 ▸). Mutations that impair ATP binding or hydrolysis have a severe effect on *in vitro* MMR activity (Tomer *et al.*, 2002 ▸; Johnson *et al.*, 2010 ▸). In addition, ATP binding is required for the interaction of MutLα with MutSα, with MLH1 predominantly being responsible for this interaction (Plotz *et al.*, 2003 ▸).

In this report, we present the X-ray crystal structure of a ternary Mg–ADP complex of the human MLH1 NTD domain determined to 2.30 Å resolution, which is the first report of a human MLH1 structure. As missense variants that disrupt the structure and/or function of this domain have the potential to cause disease, our structure helps to provide a direct mechanistic explanation to support the functional effect of *MLH1* variants identified in patients who receive clinical genetic testing.

## 2. Materials and methods   

### 2.1. Protein expression and purification   

The sequence encoding the N-terminal domain of hMLH1 (residues 1–340) was amplified by PCR and subcloned into the pET-28-MHL vector (GenBank deposition ID EF456735) downstream of the polyhistidine affinity tag. The protein was overexpressed in *Escherichia coli* BL21 (DE3) V2R-pRARE cells in Terrific Broth medium in the presence of 50 µg ml^−1^ kanamycin. The cells were grown at 37°C to an OD_600 nm_ of 1.5, induced by the addition of 1 m*M* isopropyl β-d-1-thio­galactopyranoside (IPTG) and incubated overnight at 15°C. The cells were harvested by centrifugation at 7000 rev min^−1^ and resuspended in 50 m*M* HEPES pH 7.4, 500 m*M* NaCl, 2 m*M* β-mercaptoethanol, 5% glycerol, 0.1% CHAPS, 1 m*M* phenylmethylsulfonyl fluoride (PMSF). The cells were lysed by passage through a microfluidizer (Microfluidics Corporation) at 138 MPa. After clarification of the crude extract by high-speed centrifugation, the lysate was applied onto a 5 ml HiTrap Chelating column (GE Healthcare) charged with Ni^2+^. The column was washed with ten column volumes of 20 m*M* HEPES pH 7.4 containing 500 m*M* NaCl, 50 m*M* imidazole and 5% glycerol. The protein was eluted in 20 m*M* HEPES pH 7.4, 500 m*M* NaCl, 250 m*M* imidazole, 5% glycerol and then loaded onto a Superdex 200 (26/60, GE Healthcare) column equilibrated in 20 m*M* PIPES pH 6.5 buffer containing 250 m*M* NaCl. TEV protease was added to the combined fractions containing MLH1. The protein was further purified to homogeneity by ion-exchange chromatography on a Source 30S column (10/10; GE Healthcare) and eluted in a final buffer consisting of 20 m*M* PIPES pH 6.5, 250 m*M* NaCl.

### 2.2. Crystallization and structure determination   

Purified MLH1 protein (10 mg ml^−1^) was mixed with ADP at a 1:5 molar ratio of protein:ligand and crystallized using the sitting-drop vapor-diffusion method by mixing 1 µl protein solution with 1 µl reservoir solution consisting of 20% PEG 4000, 10% 2-propanol, 0.1 *M* HEPES pH 7.5.

Diffraction data were collected on beamline 19ID at the Advanced Photon Source, Argonne National Laboratory. Reflection intensities from 150 1° diffraction images were initially integrated and scaled using *HKL*-3000 (Minor *et al.*, 2006 ▸). Using the crystal structure of *E. coli* MutL (PDB entry 1b62; 36% amino-acid sequence identity; Ban *et al.*, 1999 ▸; Johnson *et al.*, 2008 ▸) as the search model, the structure was solved by molecular replacement with *MOLREP* (Vagin & Teplyakov, 2010 ▸). The initial refinement alternated cycles of restrained refinement including TLS parameterization in *REFMAC* (Murshudov *et al.*, 2011 ▸; Winn *et al.*, 2001 ▸) with interactive rebuilding in *Coot* (Emsley *et al.*, 2010 ▸). After renewed processing of the same diffraction images with *XDS* (Kabsch, 2010 ▸) and additional scaling with *AIMLESS* (Evans & Murshudov, 2013 ▸), the model was further refined using *autoBUSTER* (Blanc *et al.*, 2004 ▸; Bricogne *et al.*, 2011 ▸) and *REFMAC* interspersed with interactive rebuilding.

The *MolProbity* statistics of the model compared favorably with a set of reference structures with similar data resolution (*MolProbity* server v.4.1-537). The model was deposited in the PDB using the *PDB_EXTRACT* tool (Yang *et al.*, 2004 ▸) with accession code 4p7a. Data-collection, model-refinement and validation statistics are summarized in Table 1 ▸. All figures were prepared using *PyMOL* (v.1.5.0.4; Schrödinger).

## 3. Results and discussion   

### 3.1. Overall structure   

**Figure 1:**
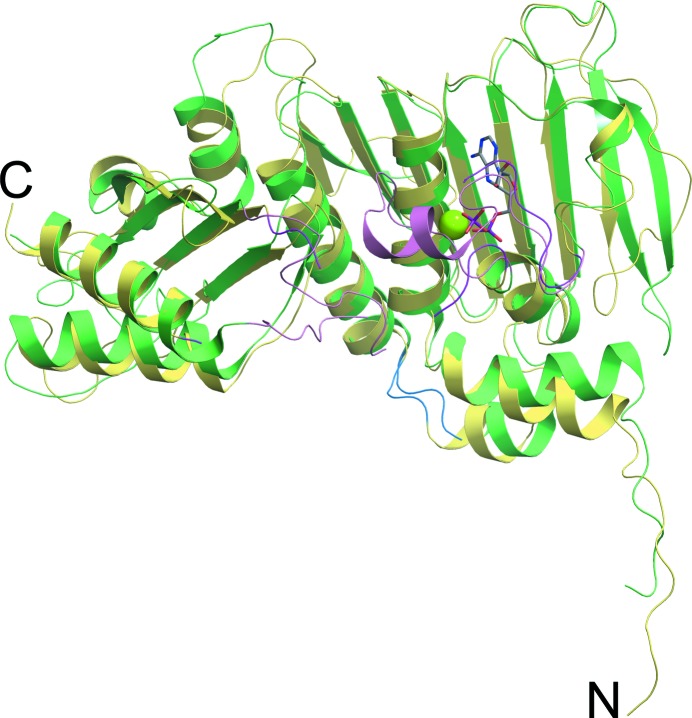
Superimposition of hLN40 and *E. coli* LN40 (PDB entry 1b62). hLN40 is colored yellow, while the *E. coli* homolog is colored green. The ATPase and transducer domains are located to the right and left, respectively, of the short loop colored blue. Residues in the ATP-binding loop of hLN40 are colored magenta, while those in *E. coli* LN40 are colored pink (the loop in the latter is ordered owing to extensive crystal contacts). In hLN40, ADP is depicted in stick representation and Mg^2+^ is shown as a green sphere. Secondary-structure elements are labelled beginning at the N-­terminus, with the first helix being αA and the first β-strand being β1.

Crystals of the hMLH1 NTD formed in space group *P*6_4_ with one molecule in the asymmetric unit. The crystallized hMLH1 construct contained residues 1–340 of the full-length protein. The crystallographic model included amino-acid residues 3–85, 98–299 and 320–336. Atoms with little or no electron density were deemed to be disordered and were omitted from the final model. Also included were ADP, an Mg^2+^ ion, 35 water molecules and nine sites with electron densities that we failed to confidently interpret in terms of specific chemical features. These sites are designated ‘UNX’ in the coordinate file (unknown atoms or ions). A *DALI* search (Holm & Rosenström, 2010 ▸) identified the *E. coli* MutL NTD (LN40; Ban & Yang, 1998 ▸) as the closest structural homolog (Fig. 1 ▸). Superimposition of our structure with the *E. coli* MutL–Mg–ADP ternary complex (PDB entry 1b62) using *CEAlign* (Jia *et al.*, 2004 ▸; Shindyalov & Bourne, 1998 ▸) matches 288 C^α^ positions with a root-mean-square deviation (r.m.s.d.) of 2.5 Å. Given the similarity to *E. coli* MutL NTD and to be consistent with the nomenclature established by Ban & Yang (1998 ▸), we designate our structure human LN40 (hLN40).

The overall structure of hLN40 can be divided into two subdomains (Fig. 1 ▸), an ATPase domain and a ‘transducer’ domain, connected by a two-helix linker. The ATPase domain (residues 25–207) contains the noncanonical, ATPase Bergerat fold, the core of which is composed of a four-stranded, antiparallel β-sheet (β1–β3 and β5) and three α-helices (αB–αD) (Bergerat *et al.*, 1997 ▸). The fold is essentially identical to the topology observed in *E. coli* LN40 and identifies MLH1 as a member of the GHKL (**g**yrase, **H**sp90, histidine **k**inase, Mut**L**) ATPase/kinase superfamily of proteins (Dutta & Inouye, 2000 ▸). The ATP-binding loop between helices αC and αD (residues 74–85 and 98–101) defines the pyrophosphate binding site and is variable in structure and length across the family (Ban *et al.*, 1999 ▸; Prodromou *et al.*, 1997 ▸; Steussy *et al.*, 2001 ▸; Wigley *et al.*, 1991 ▸). In addition to the similarity observed in the overall structure between hLN40 and the MutL structure (Ban *et al.*, 1999 ▸), we also observed the presence of an hLN40 crystallographic dimer similar to that observed in the *E. coli* MutL–Mg–ADP complex. However, in contrast to the prokaryotic structure, the hLN40 ATP-binding loop is partially disordered, possibly owing to crystal packing. Accordingly, residues 86–97 have been omitted from our model owing to a lack of interpretable electron density. The C-terminus of the ATP-binding loop is part of a conserved GFRGE(A/G)L motif (residues 98–104) that is found in related mismatch-repair proteins (Sehgal & Singh, 2012 ▸) and is an extension of motif III (the ‘G2 box’) conserved in GHKL family members (Mushegian *et al.*, 1997 ▸). Gly98 and Gly101 are positioned adjacent to the pyrophos­phate moiety of the bound ADP, permitting the close approach of ADP to the N-terminus of helix αD. This allows the negatively charged ligand to take advantage of a half positive unit charge that arises from the helix dipole moment (Hol *et al.*, 1978 ▸; Wierenga *et al.*, 1985 ▸). The presence of a glycine-rich motif is consistent with a conserved mechanism that has evolved to play a crucial role in the active site of several nucleotide-binding folds (Saraste *et al.*, 1990 ▸; Walker *et al.*, 1982 ▸; Wierenga *et al.*, 1985 ▸).

Residues 228–336 fold separately to form a small α/β barrel at the hLN40 C-terminus, known as the transducer domain (Classen *et al.*, 2003 ▸). This domain is characterized by a ribosomal protein S5 domain 2-like fold (Murzin *et al.*, 1995 ▸) and a left-handed α-helical crossover (αI) between β10 and β11 (Ban *et al.*, 1999 ▸; Cole & Bystroff, 2009 ▸; Richardson, 1976 ▸). A large body of evidence points towards the allosteric regulation of the transducer domain playing a central role in coordinating the downstream functions of GHKLs (Ban *et al.*, 1999 ▸; Corbett & Berger, 2003 ▸, 2005 ▸; Lamour *et al.*, 2002 ▸; Oestergaard *et al.*, 2004 ▸; Wei *et al.*, 2005 ▸; Wigley *et al.*, 1991 ▸). In particular, the ‘QTK’ loop (hLN40 residues 298–320) has been proposed to act as an ATP ‘sensor’ that helps to couple changes in ligand binding and hydrolysis to rigid-body movements and conformational changes in the transducer domain (Wei *et al.*, 2005 ▸). Residues 301–320 in the hLN40 QTK loop are disordered; however, we can infer from MutL structures (Ban *et al.*, 1999 ▸) that Lys311 within the PTK motif should act as the conserved basic, γ-phosphate-sensing residue. Crystallographic studies by both Corbett & Berger (2005 ▸) and Stanger *et al.* (2014 ▸) highlight the importance of rigid-body motions between the ATPase and transducer domains of GHKLs. In particular, these studies identified several distinct conformational intermediates that exist along the ATP-hydrolysis pathway. However, without further structural and biochemical information on catalytically competent forms of hLN40, it remains to be seen whether these observations represent a unifying mechanism that explains how GHKLs achieve their higher-order functions in the cell.

### 3.2. Structural basis for the pathogenicity of *MLH1* mutations   

**Figure 2:**
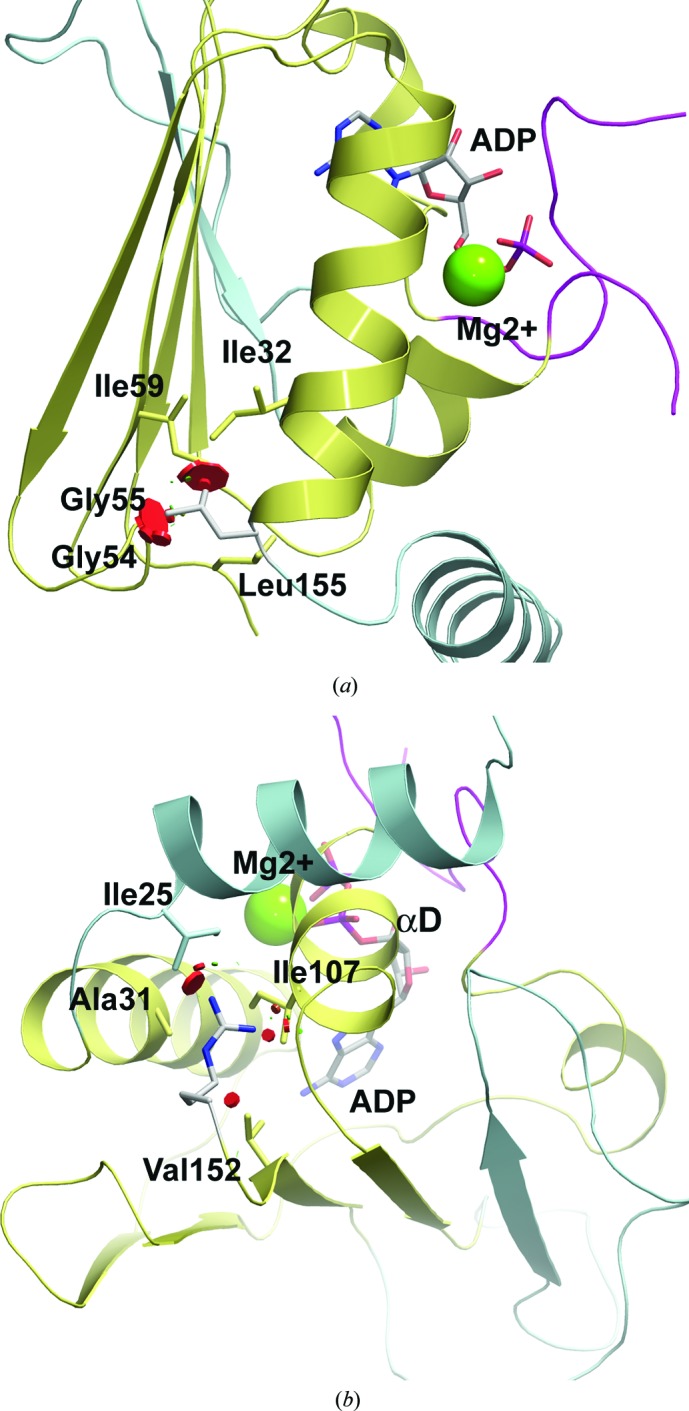
Structural basis for the pathogenicity of *MLH1* missense variants. Ribbon diagrams showing the structural consequences of (*a*) c.83C>T (p.Pro28Leu) and (*b*) c.464T>G (p.Leu155Arg). The figure is colored as in Fig. 1 ▸, with the exception that structural elements outside the core Bergerat fold are colored cyan. Important amino acids around the mutation are represented as sticks. The mutation is colored grey. Red circles represent steric clashes with surrounding parts of the structure. For clarity, the transducer domain is omitted from both figures.

Structural and functional information may be utilized to determine the pathogenicity of *MLH1* mutations identified during genetic testing for hereditary cancer syndromes. Here, we present two such pathogenic variants, c.83C>T (p.Pro28Leu) and c.464T>G (p.Leu155Arg) (Thompson *et al.*, 2014 ▸). Pro28 is a buried residue at the N-terminus of αA in the ATPase domain and is completely inaccessible to the solvent (Krissinel & Henrick, 2007 ▸). The introduction of a Leu at this tightly packed position in p.Pro28Leu is likely to introduce severe steric clashes, given its more extended side chain. Sterically, the most favorable rotamer still shows increased van der Waals (vdW) strain and steric clashes involving Gly54, Gly55, Ile59 and Ile176 that are likely to disrupt the core fold of the protein (Fig. 2 ▸ *a*).

Leu155 is also buried in the α/β sandwich of the ATPase domain, between helix αB and the extended β-sheet (Fig. 2 ▸ *b*). Substitution by Arg at this position could have two consequences. Firstly, outside an active site or stabilizing secondary-structure element, the introduction of an unbalanced, buried charge is often considered to be destabilizing to protein structure (Kajander *et al.*, 2000 ▸; Waldburger *et al.*, 1995 ▸; Wimley *et al.*, 1996 ▸). Incorporating the most favorable rotamer, the modeled Arg at position 155 is surrounded by a cluster of nonpolar residues (Ala31, Ile25, Ile107 and Val152) and is unable to form hydrogen bonds to nearby side-chain or main-chain atoms. The second structural consequence of p.Leu155Arg relates to the compact space in the center of the α/β sandwich, which imposes a steric constraint on the type of amino acid that can be accommodated at position 155. Compared with Leu, the more extended alkyl-guanidinium side chain of Arg introduces severe steric clashes, which disrupt the architecture of the elements (for example helix αD) that form the active site of the enzyme.

Given this structural rationale, we expect the MLH1 structure reported here to be of great clinical utility in the analysis of missense variants found in patients recommended for genetic testing. The structure provides a robust platform, in combination with other strong functional or clinical evidence, to help to determine the clinical effect of loss-of-function mutations. We caution, however, against reliance on this model to predict a benign effect in a clinical setting, as truly pathogenic variants may fall within the ‘normal’ functional range. Therefore, other factors must be considered when a seemingly benign substitution is encountered, including the possibility that a nonsynonymous change may have an effect on mRNA splicing or post-translational modification of the protein.

## Supplementary Material

PDB reference: human MLH1 N-terminus, 4p7a

## Figures and Tables

**Table 1:** Data-collection, refinement and validation statistics for the hLN40 structure

Data collection/reduction	
Radiation source	19ID, APS
Wavelength ()	0.9793
Space group	*P*6_4_
Unit-cell parameters (, )	*a* = *b* = 94.57, *c* = 85.82, = = 90.00, = 120.00
Resolution limits ()	47.282.30 (2.382.30)
Unique reflections	19468 (1888)
Completeness (%)	99.9 (100.0)
*R*_merge_	0.059 (1.11)
*R*_meas_	0.062 (1.18)
Mean *I*/(*I*)	27.1 (2.3)
Multiplicity	9.5 (9.5)
Model refinement	
Resolution ()	40.002.30
Reflections used/in test set	18456/981
No. of atoms	
Total	2296
Protein	2222
Water	37
Others	37
Average *B* factor (^2^)	
Overall	65.9
Protein	66.7
Water	45.9
Others	39.9
Wilson *B* factor† (^2^)	51.4
*R*_work_/*R*_free_	0.203/0.254
R.m.s.d., bonds ()/angles ()	0.014/1.4
Model validation‡	
Ramachandran plot	
Favored (%)	98.33
Outliers (%)	0.00
Clashscore	1.82
*MolProbity* score	1.15

† Obtained using *phenix.model_vs_data* (Afonine *et al.*, 2010 ▸).

‡ Obtained using *phenix.molprobity* (Adams *et al.*, 2010 ▸; Chen *et al.*, 2010 ▸).
